# Transcriptional profiling of the mouse hippocampus supports an NMDAR‐mediated neurotoxic mode of action for benzo[*a*]pyrene

**DOI:** 10.1002/em.22020

**Published:** 2016-05-19

**Authors:** Nikolai L. Chepelev, Alexandra S. Long, Wayne J. Bowers, Rémi Gagné, Andrew Williams, Byron Kuo, David H. Phillips, Volker M. Arlt, Paul A. White, Carole L. Yauk

**Affiliations:** ^1^ Environmental Health Science and Research BureauEnvironmental and Radiation Health Sciences Directorate, HECSB, Health CanadaOttawaOntarioCanadaK1A 0K9; ^2^ Analytical and Environmental Sciences DivisionMRC‐PHE Centre for Environment and Health, King's College LondonLondonSE1 9NHUnited Kingdom

**Keywords:** toxicogenomics, human health risk assessment, mode of action, learning and memory, glutamate receptor

## Abstract

Benzo[*a*]pyrene (BaP) is a genotoxic carcinogen and a neurotoxicant. The neurotoxicity of BaP is proposed to arise from either genotoxicity leading to neuronal cell death, or perturbed expression of *N*‐methyl‐d‐aspartate receptor (NMDAR) subunits. To explore these hypotheses, we profiled hippocampal gene expression of adult male Muta^™^Mouse administered 0, 1, 35, or 70 mg BaP/kg bw per day by oral gavage for 3 days. Transcriptional profiles were examined by RNA‐sequencing (RNA‐seq), DNA microarrays, and real‐time quantitative reverse transcription polymerase chain reaction (RT‐PCR). BaP‐DNA adducts in the cerebellum were quantified by ^32^P‐post‐labeling to measure genotoxicity. RNA‐seq revealed altered expression of 0, 260, and 219 genes (*P*‐value < 0.05, fold‐change ≥ ± 1.5) following exposure to the low, medium, and high doses, respectively; 54 genes were confirmed by microarrays. Microarray and RT‐PCR analysis showed increased expression of NMDAR subunits *Grina* and *Grin2a*. In contrast, no effects on DNA‐damage response genes were observed despite comparable BaP‐DNA adduct levels in the cerebellum and in the lungs and livers of mice at similar BaP doses in previous studies. The results suggest that DNA‐damage response does not play a major role in BaP‐induced adult neurotoxicity. Meta‐analysis revealed that BaP‐induced transcriptional profiles are highly correlated with those from the hippocampus of transgenic mice exhibiting similar neurotoxicity outcomes to BaP‐exposed mice and rats (i.e., defects in learning and memory). Overall, we suggest that BaP‐induced neurotoxicity is more likely to be a consequence of NMDAR perturbation than genotoxicity, and identify other important genes potentially mediating this adverse outcome. Environ. Mol. Mutagen. 57:350–363, 2016. © 2016 Her Majesty the Queen in Right of Canada. Environmental and Molecular Mutagenesis © 2016 Environmental Mutagen Society.

## INTRODUCTION

Benzo[*a*]pyrene (BaP)^1^ is a human carcinogen that operates through a genotoxic mode of action (MOA) [IARC, International Agency for Research on Cancer, 2010]. In addition to causing tumors at multiple sites in rodent cancer bioassays, rodents exposed to BaP exhibit poor performance in behavioral tests, suggesting that BaP retards learning and memory in laboratory animals (reviewed in [Chepelev et al., 2015]). For example, oral exposure of neonate pups to BaP for six days leads to a significant decrease in Morris water maze test performance measured 63 days after the last exposure [Chen et al., 2012].

Laboratory animals exposed to BaP in utero, postnatally, and during adulthood exhibit neurotoxicity at doses below those inducing carcinogenicity (reviewed by [Moffat et al., 2015]). Benchmark dose (BMD) modeling of neurotoxicity and carcinogenicity in rodent experiments supports this [Chepelev et al., 2015]. In addition, several epidemiological studies have revealed potential neurotoxic effects of BaP (as a part of ambient mixtures of polycyclic aromatic hydrocarbons [PAHs]) in humans. For example, exposure to PAH mixtures was associated with a drop in IQ of up to 4.9 points in children 5 years of age [Perera et al., 2009]. A drop in IQ of similar magnitude to that observed following BaP exposure in PAH mixtures is also elicited by other well‐characterized neurotoxicants. For instance, an IQ drop of 1–3 is associated with exposure to arsenic in 5‐year‐old girls [Hamadani et al., 2011]. In addition, there is an association between “PAH‐like” DNA adducts in children and adverse cognitive/behavioral development [Perera et al., 2011; Perera et al., 2015]. Given these observed neurotoxic effects of BaP at low doses, and their apparent human relevance, the mechanisms underlying BaP neurotoxicity in both developing and adult exposure scenarios merit further investigation.

**Figure 1:**
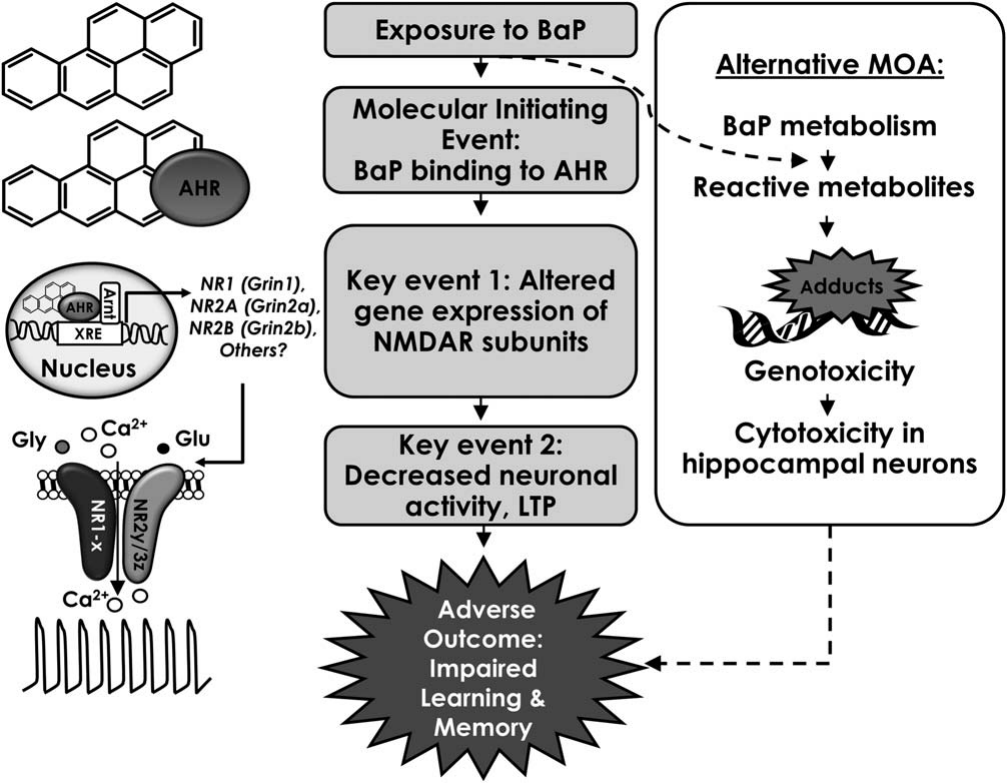
Proposed mode of action (MOA) for BaP‐induced neurotoxicity in rodents (reviewed in and redrawn from [Chepelev et al., 2015]). The MOA for BaP neurotoxicity in rodents is hypothesized to proceed though the altered expression of *N*‐methyl D‐aspartate receptors (NMDARs) that are heterotetramers of two obligatory glycine‐binding NR1 (a.k.a. Glutamate receptor, ionotropic, *N*‐methyl D‐aspartate 1 or Grin1) subunits and regulatory glutamate‐binding NR2 (Grin2) and/or NR3 (Grin3) subunits. The altered expression of NMDARs in response to BaP has been shown in several studies [Brown et al., 2007; Grova et al., 2007; Grova et al., 2008; Qiu et al., 2011; Cheng et al., 2013] and could be due to transcriptional regulation of xenobiotic response element‐ (XRE‐) containing genes (e.g., *Grin2a*) by aryl hydrocarbon receptor (AHR), following its binding to BaP, nuclear translocation, and heterodimerization with AHR nuclear translocator (Arnt). Altered hippocampal expression of NMDAR may alter the magnitude of NMDAR‐mediated ion currents, resulting in reduced long‐term potentiation (LTP) and, hence, impaired learning and memory. An alternative MOA involves the genotoxicity of BaP metabolites, which could lead to neuronal death and presumably similar behavioral outcome.

A detailed review of ∼40 published studies on BaP‐induced neurotoxicity [Chepelev et al., 2015] suggested that the most well‐supported MOA to explain the neurotoxicity of BaP involves the following sequential key events: (1) BaP binding to the aryl hydrocarbon receptor (AHR); (2) altered transcription of *N*‐methyl‐d‐aspartate glutamate receptor (NMDAR) subunits due to AHR activation; (3) NMDAR‐mediated decreases in neuronal activity and long‐term potentiation; and (4) compromised learning and memory (Fig. 1). Although the specific underlying mechanisms of BaP neurotoxicity may be different in developing animals and adults, perturbed NMDAR expression has been reported in both postnatal [Brown et al., 2007; Patri et al., 2013] and adult mice and rats [Grova et al., 2007; Grova et al., 2008; Qiu et al., 2011; Cheng et al., 2013], and even in rat cortical neurons [Brown et al., 2007] and neuroblastoma Neuro2a cells [Patri et al., 2013] following BaP exposure. Thus, this proposed MOA may be relevant to both adult and in utero/postnatal exposure to BaP.

NMDARs are heterotetramers composed of two obligatory glycine‐binding NR1, or Grin1 (Glutamate receptor, ionotropic, *N*‐methyl D‐aspartate 1) subunits, and regulatory glutamate‐binding NR2 (Grin2) and/or NR3 (Grin3) subunits [Stephenson, 2006]. Alterations in NMDAR subunit expression have significant effects on learning and memory in mice and rats. Specifically, conditional knock‐out of NR1 in mouse hippocampus, the site where the conversion of short‐term to long‐term memories takes place, leads to impaired ability to learn the location of the hidden platform in the Morris water maze test [Shimizu et al., 2000]. In contrast, overexpression of NR2B subunits in the mouse forebrain increases the duration of the NMDAR currents and significantly improves the performance of mice in various behavioral tasks [Tang et al., 2001]. The importance of NMDARs in maintaining functional learning and memory in rodents has prompted researchers to examine NMDAR gene expression during BaP‐induced neurotoxicity in rats and mice [Brown et al., 2007; Grova et al., 2007; Grova et al., 2008; Qiu et al., 2011; Cheng et al., 2013].

In addition to NMDAR‐mediated neurotoxicity, an intriguing hypothesis has emerged that genotoxic compounds may cause both carcinogenicity and neurotoxicity, depending upon the presence (e.g., hepatocytes) or absence (e.g., post‐mitotic neurons) of active cell division [Kisby et al., 2011]. A good example of such a compound is methylazoxymethanol (MAM). MAM reduces cell viability in the cerebellar granule neurons [Kisby et al., 2009], most likely as a result of DNA damage (reviewed in [Kisby et al., 2013]). MAM causes both colon cancer and neurotoxicity, and perturbs similar cellular signaling pathways in these tissues [Kisby et al., 2011]. The three pathways that are shared between MAM‐induced colon cancer and neurological diseases are the transforming growth factor‐beta (TGF‐beta) pathway, the wingless and proto‐oncogene Int‐1 (Wnt) pathway, and mitogen‐activated protein kinase (MAPK) signaling pathways [Kisby et al., 2011]. Whether these pathways or other downstream consequences of BaP genotoxicity in the brain are shared between cancer and neurotoxicity rodent models in general, and between BaP‐mediated carcinogenicity and neurotoxicity in particular, is unknown.

We have extensively investigated the mutagenic effects of acute and subchronic (i.e., 3‐ or 28‐day) oral exposure to BaP in several tissues of adult Muta^™^Mouse transgenic rodents (e.g., [Moffat et al., 2015; Long et al., 2016; Moffat et al., 2015]). Since BaP is a well‐recognized mutagenic carcinogen, the results showed expected perturbations in the genes involved in the p53‐mediated DNA damage response pathway, including cyclin‐dependent kinase inhibitor 1A (*Cdkn1a*), following acute exposures that are anchored to measured genotoxic effects (reviewed in [Moffat et al., 2015]). Therefore, it is reasonable to speculate that cytotoxic and genotoxic effects of BaP on the hippocampus contribute to the observed neurotoxic effects. Transcriptomics studies can be applied to evaluate this hypothesis as an alternative MOA for BaP neurotoxicity (Fig. 1), and our previous work supports that transcriptional changes are measurable indicators of genotoxicity.

In the present study we examined global hippocampal transcriptional profiles of adult Muta^TM^Mouse males acutely exposed to BaP in order to: (1) test the hypothesis that the neurotoxic effects of BaP in adult rodents are predominantly manifested via the modulation of NMDAR expression (i.e., versus DNA damage response leading to increased neuronal cell death); (2) identify potentially shared signaling pathways for BaP‐induced genotoxicity and neurotoxicity; and (3) explore other plausible MOAs or modulating factors involved in BaP‐induced neurotoxicity.

## MATERIALS AND METHODS

### Animal Treatment

Adult Muta™Mouse (strain 40.6, 9 weeks of age) males were individually housed in a microVENT ventilated rack (Allentown Inc., Allentown, NJ) on a 12 hr light/12 hr dark cycle. Muta™Mouse was used in this project due to the established expertise of our group in the use of this model system while studying BaP mutagenicity. Mice received standard rodent chow (2014 Teklad Global standard rodent diet) ad libitum for the duration of the study. Animals were administered BaP (CAS no. 50‐32‐8, purity ≥ 98%, Cambridge Isotopes, Tewksbury, MA) dissolved in highly refined olive oil (Sigma‐Aldrich, Oakville, ON, Canada) delivered at 0.005 ml/g body weight. BaP (0, 1, 35, and 70 mg/kg bw per day) was administered by oral gavage for three consecutive days. Doses were selected based on a previous study [Chen et al., 2012] that we determined to be the most suitable for health risk assessment [Chepelev et al., 2015] and that used the relevant route of exposure (i.e., oral gavage). This study described significant effects on Morris water maze test performance in rats dosed postnatally (days 5‐11) with 0.2 and 2 mg/kg bw per day by oral gavage for six days (effects observed up to 63 days after the last exposure) [Chen et al., 2012]. Because we are using an adult model, we increased the lowest dose showing neurotoxicity in Chen et al. (i.e., 0.2 mg/kg bw per day) to 1.0 mg/kg bw per day to compensate for the anticipated lower susceptibility of the adult brain to BaP. We chose to focus on adult rather than developmental exposure because of the numerous adult studies demonstrating BaP‐induced neurotoxicity (e.g., [Grova et al., 2007; Grova et al., 2008; Qiu et al., 2011; Cheng et al., 2013]) and for comparison with our previous global gene expression analyses in Muta™Mouse lung [Halappanavar et al., 2011] and liver [Moffat et al., 2015] following 3‐day exposures to BaP, where we characterized extensive evidence of DNA damage response in the transcriptome.

There were five animals in each dose group, as well as in the vehicle control group. After 24 hr, animals were anesthetized with isoflurane gas and then euthanized by cardiac puncture, followed by cervical dislocation and chest cavity opening. This time interval was chosen based on a previous study showing that administration of radiolabeled BaP leads to a peak of radioactivity in the brain at 23 hr [Das et al., 1985]. The whole brain was removed, and the hippocampus and cerebellum separated. Tissues were flash‐frozen in liquid nitrogen and stored at –80°C. Mice were bred, maintained, and treated in accordance with the Canadian Council for Animal Care Guidelines. Animal care and handling were approved by Health Canada's Animal Care Committee.

### DNA Adduct Analysis

While aligning transcriptomic and DNA adduct data would be of great relevance to this study, the small amount of DNA recoverable from the mouse hippocampus (i.e., the tissue that would be the most relevant to learning and memory impairments induced by neurotoxicants) precluded DNA adduct analysis in this target tissue. Therefore, cerebellum was used as a surrogate tissue for hippocampus. Approximately half of the entire cerebellum (one hemisphere chosen randomly) was minced and combined with 5‐ml ice cold lysis buffer (1 mM Na_2_EDTA, 100 mM NaCl, 20 mM Tris‐HCl, pH 7.4) supplemented with 1% SDS (w/v) and 0.1 mg/ml RNase A, and incubated at 37˚C overnight with gentle shaking. Genomic DNA was isolated using a phenol/chloroform extraction procedure described previously [Douglas et al., 1994; Vijg and Douglas, 1996]. Isolated DNA was dissolved in 100 µl TE buffer (10 mM Tris pH 7.6, 1 mM EDTA) and stored at 4˚C until use.

DNA adducts were measured for each DNA sample using the ^32^P‐post‐labeling method using the nuclease P1 digestion enrichment version of the assay [Phillips and Arlt, 2014]. For analysis, DNA samples (4 µg) were digested with micrococcal nuclease (288 mUnits) and spleen phosphodiesterase (1.2 mUnits), enriched, and labeled as reported elsewhere [Phillips and Arlt, 2014; Arlt et al., 2015; Krais et al., 2016]. Chromatographic conditions for thin‐layer chromatography (TLC) on polyethyleneimine‐cellulose (PEI‐cellulose) plates (Macherey‐Nagel, Düren, Germany) were: D1, 1.0 M sodium phosphate, pH 6; D2, 4 M lithium‐formate, 7 M urea, pH 3.5; and D3, 0.8 M lithium chloride, 0.5 M Tris, 8.5 M urea, pH 8. After chromatography, TLC sheets were scanned using a Packard Instant Imager (Dowers Grove, IL), and DNA adduct levels (RAL, relative adduct labeling) were calculated from adduct cpm, the specific activity of [γ‐^32^P]ATP, and the amount of DNA (pmol of DNA‐P) used. An external BaP‐7,8‐dihydro‐diol‐9,10‐epoxide (BPDE)‐DNA standard was used for identification of BaP‐DNA adducts as described [Arlt et al., 2008; Krais et al., 2016]. Results are expressed as DNA adducts/10^8^ nucleotides.

### Global Transcriptional Profiling

RNA was isolated, hybridized, and analyzed as previously described [Malik et al., 2013; Jackson et al., 2014]. For RNA‐sequencing, 4 µg of RNA with RNA integrity numbers above 7.0 per sample (Agilent 2100 Bioanalyzer) was shipped on dry ice to the McGill University and Génome Québec Innovation Centre (Montréal, Canada), where cDNA libraries were built following the polyA‐enrichment Illumina TruSeq v2 protocol. Hippocampi sample cDNA libraries were then ligated to Illumina adapters and paired‐end sequenced on a HiSeq2000 with N = 5 for control, N = 4 for low, medium, and high doses. Sequencing was performed at the McGill University and Génome Québec Innovation Centre (Montréal, Canada). The sequencing depth was 40 million reads and the read length was 100 base pairs.

Data processing was carried out with Illumina's Real Time Analysis software, and converted into FASTQ files using Illumina's CASAVA software. The GRCm38 mouse genome was used to align the FASTQ files with STAR's default parameters [Dobin et al., 2013]. Feature counting of the Ensembl Gene Set (GRCm38v75) was performed in Python using HTSeq‐count (version 0.6.1) [Anders et al., 2015]; the m parameter was set to “intersection‐nonempty.” The EdgeR [Robinson et al., 2010] and TMM normalization [Robinson and Oshlack, 2010] were used to calculate differentially expressed genes using the exactTest function, producing gene lists with fold‐change with respect to controls and the corresponding false‐discovery rate‐ (FDR) adjusted p‐values. The complete dataset is available through the Sequence Read Archive (SRA) at http://www.ncbi.nlm.nih.gov/sra, project number PRJNA304295.

For microarray analysis, cDNA and cyanine‐labeled cRNA were synthesized (Agilent Linear Amplification Kit) from 250 ng of total RNA from sample and universal mouse reference total RNA (Stratagene, Canada). Samples were in vitro transcribed and hybridized using a two‐color reference design as described previously [Labib et al., 2012] to Agilent 8x60 K whole genome microarrays. Arrays were scanned on an Agilent G2505B scanner and data acquired using Agilent Feature Extraction Software, version 11. Normalization was performed using locally weighted scatterplot smoothing (LOWESS) [Yang et al., 2002] in R [R‐Development‐Core‐Team, 2010]. The determination of differential gene expression was carried out by microarray analysis of variance (MAANOVA) [Wu et al., 2003], and the false‐discovery rate‐ (FDR) adjusted p‐values were calculated as described elsewhere [Jackson et al., 2014]. The complete dataset is available from GEO (accession number GSE75206).

### Real‐Time Quantitative Reverse Transcription Polymerase Chain Reaction (RT‐PCR) Validation

A custom RT‐PCR array containing proprietary probes (Qiagen) was designed to measure the expression of certain NMDARs that were differentially expressed in previous studies in response to BaP in the brain, to test our hypothesis of the involvement of NMDARs in mediating BaP neurotoxicity, as well as to validate the RNA‐seq and microarray data. Therefore, the arrays measured the expression of *Grin1*, *Grin2a*, and *Grin2b* (at least one of these genes was affected in previous studies [Grova et al., 2007; Grova et al., 2008; Qiu et al., 2011; Cheng et al., 2013]), other NMDAR genes (*Grina* and *Grin3a*), and some genes identified as differentially expressed by our RNA‐seq and/or microarray analysis: claudins 2 and 5 (*Cldn2* and *Cldn5*), ephrin type‐A receptor 7 (*Epha7*), hypoxia‐inducible factor 3A (*Hif3a*), 3‐hydroxy‐3‐methylglutaryl‐CoA synthase 2 (*Hmgcs2*), perilipin 4 (*Plin4*), semaphorin‐3B (*Sema3b*), and semaphorin‐5A (*Sema5a*). In addition, the arrays contained Glucuronidase beta (*Gusb*) to act as housekeeping gene for normalization as in previous work [Labib et al., 2012], and mouse cDNA genomic contamination control and reverse transcription control reactions. Total RNA that isolated from the hippocampus during sample preparation for RNA‐seq and microarray analyses was used. RT^2^ Easy First Strand Kits (Qiagen) were used to generate cDNA libraries from 0.5 micrograms of total RNA, in accordance with the manufacturer's protocol. Arrays were run on a CFX96 real‐time detection system (Bio‐Rad). C_t_ values were normalized to *Gusb* and gene expression was analysed at http://pcrdataanalysis.sabiosciences.com/pcr/arrayanalysis.php.

### Bionformatic Analyses

All genes with FDR *P* ≤ 0.05 and fold change ≥ ± 1.5 relative to controls were analysed to identify biological pathways, functions, or processes that are affected in the hippocampus following BaP exposure by Ingenuity Pathway Analysis™ (IPA™, Ingenuity Systems, Redwood City, CA) and NextBio™ (http://nextbio.com). Fisher's exact tests were used in IPA to examine the significance of the association between a given dataset and IPA‐defined canonical pathways and functions. NextBio™ was used to compare a given dataset to their library of curated datasets to identify studies with similar gene expression profiles using the meta‐analysis function. The significance of negative or positive correlations between datasets is assessed in NextBio™ by considering a number of factors, most notably, directionality and the strength of the overlap/enrichment between a pair of datasets [Kupershmidt et al., 2010].

### Statistical Analyses

The DNA adduct data were analysed using SAS software, Version 9.1 of the SAS System for Windows (© 2013 SAS Institute Inc, Cary, NC) by Poisson regression. The data were fitted to the model log(E(Yi)) = log ti + βxi, where E(Yi) is the expected value for the ith observation, β is the vector of regressions coefficients, xi is a vector of covariates for the ith observation, and ti is the offset variable used to account for differences in observation count period. The offset was given a constant coefficient of 1.0 for each observation, and log‐linear relationships between adduct frequency and test article concentration were specified by a natural log link function. Type 1, or sequential analysis, was employed to examine the statistical significance of the chemical treatment. Body weights were analysed by the “ANOVA” (one‐way analysis of variance) function in Microsoft Excel.

### Benchmark Dose (BMD) Modeling

For RNA‐seq data, normalized log2‐transformed expression values for individual genes were fit as continuous data to a series of four dose‐response models (Hill, power, linear, and second degree polynomial) by BMDExpress software [Yang et al., 2007]. Each model was run assuming constant variance, with maximum iterations of 250, benchmark response (BMR) factor of 1.349, confidence level of 0.95, and power restricted to ≥ 1. For the Hill model, if the k parameter was less than 1/3 of the lowest dose, the output was flagged, and the next best fit model was selected if it had a goodness‐of‐fit *P*‐value > 0.05. The probes were filtered (ANOVA *P*‐value ≤ 0.05) prior to the analysis, as described elsewhere [Webster et al., 2015], and BMD analysis was performed only on differentially expressed probes.

## RESULTS

### General Response of Muta™Mouse to BaP

Mice treated with 1, 35, or 70 mg/kg bw BaP per day for 3 days displayed neither overt signs of toxicity, nor body weight loss relative to controls (data not shown). Serum biochemistry tests revealed increases in lipase and decreases in magnesium at all of the doses compared to controls, and an increase in creatinine at the medium dose (Supporting Information Fig. 1).

#### DNA Adducts in the Cerebellum

**Figure 2:**
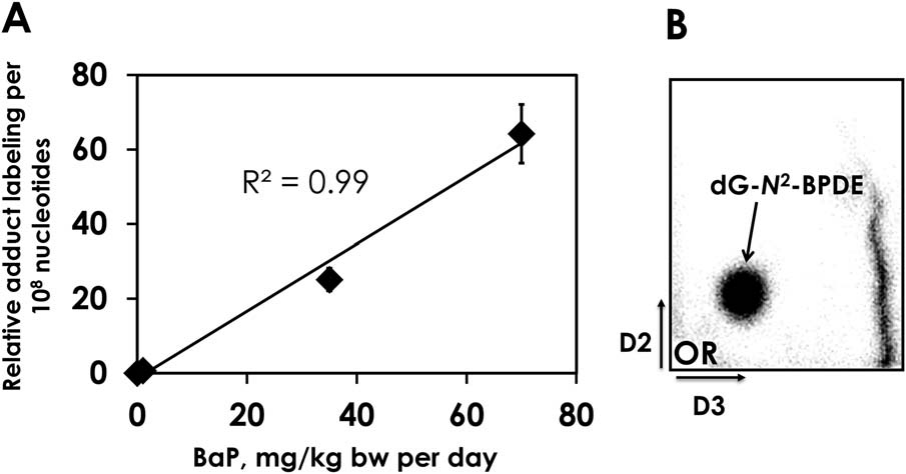
Dose‐dependent formation of BaP‐DNA adducts detected in the cerebellum of Muta™Mouse by ^32^P‐post‐labeling. **A**: Error bars represent standard error of the mean of four to five mice (each DNA sample analyzed in two independent post‐labeling assays). Asterisks (*) denote statistically significant differences from control at the *P* < 0.0001 level. **B**: Representative autoradiographic adduct profile obtained by ^32^P‐post‐labeling in the cerebellum of Muta™Mouse dosed with BaP. The major spot shows 10‐(deoxyguanosin‐*N* ^2^‐yl)‐7,8,9‐trihydroxy‐7,8,9,10‐tetrahydro‐BaP (dG‐*N* ^2^‐BPDE). The origin (OR), at the bottom left‐hand corner, was cut off before exposure. D2 and D3 indicate the solvent conditions used on the TLC (see Materials and Methods).

Analysis of cerebellar DNA showed dose‐dependent increases in BaP‐DNA adducts, with significant increases in adduct levels at all concentrations relative to control (Fig. 2A). The DNA adduct pattern observed by TLC ^32^P‐post‐labeling in BaP‐treated mice consisted of a single adduct spot that was previously identified by mass spectrometry as 10‐(deoxyguanosin‐*N* ^2^‐yl)−7,8,9‐trihydroxy‐7,8,9,10‐tetrahydro‐BaP (dG‐*N* ^2^‐BPDE) [Arlt et al., 2008] (Fig. 2B). No adduct formation was detected in cerebellar DNA isolated from untreated mice (data not shown). The highest adduct level detected was 64.2 ± 17.6 adducts per 10^8^ nucleotides at the highest dose (Fig. 2).

**Figure 3:**
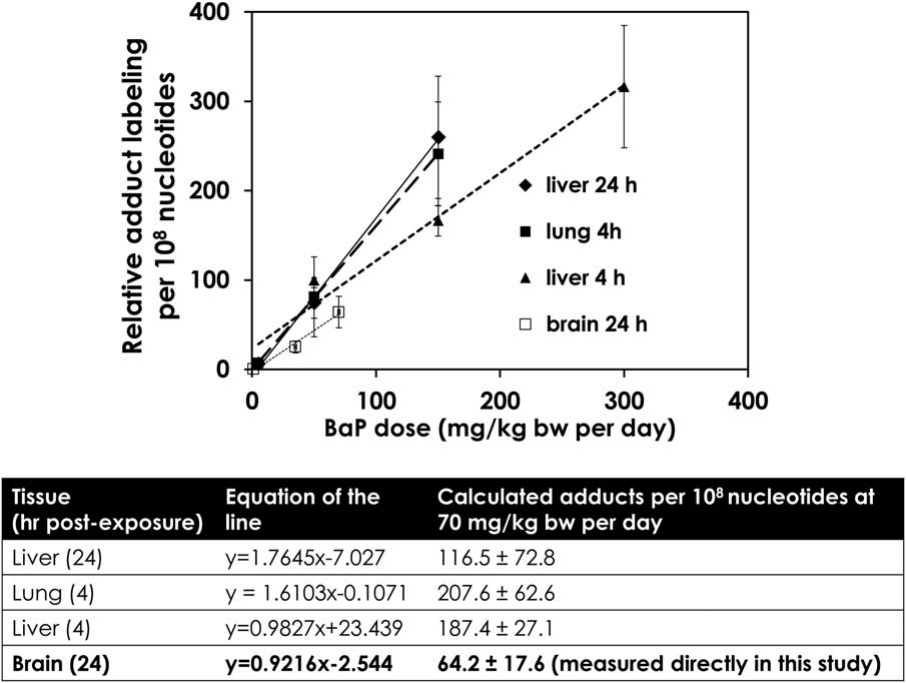
Dose‐response comparison of BaP‐DNA adduct levels detected by ^32^P‐postlabeling in lung, liver ([Halappanavar et al., 2011] and unpublished data), and cerebellum (this study) in Muta^TM^Mouse dosed with BaP for three consecutive days by oral gavage. Plotted values are means ± standard deviation (*n* = 8). Estimated adduct levels for lung and liver at the high dose used in this study (i.e., 70 mg BaP/kg bw per day) are shown in the table. Estimates are based on linear trends (i.e., slopes of dose‐response function) ± standard error of the interpolated values that were calculated from the mean values plotted here.

We compared our findings to adduct data from lung and liver samples in a previous study on Muta™Mouse males treated with 0, 5, 50, 150, and 300 mg/kg bw BaP per day for 3 days [Halappanavar et al., 2011]. These authors also noted a dose‐dependent increase in the levels of adducts in the lungs and livers of exposed animals. Interpolation from this dose‐response curve suggests that the BaP‐DNA adduct levels in Muta™Mouse lung and liver at 70 mg/kg bw per day exposure would be 1.8 − 3.2 times higher than the adduct levels observed at this exposure level in the cerebellum in this study. However, the interpolated value for the liver measured 24 h following BaP exposure is 116.5 ± 72.8 adducts per 10^8^ nucleotides, which overlaps with the cerebellum adduct level of 64.2 ± 17.6 adducts per 10^8^ nucleotides from this study at 70 mg/kg bw per day (Fig. 3).

#### Gene Expression Changes in the Hippocampus by RNA‐Sequencing

**Figure 4:**
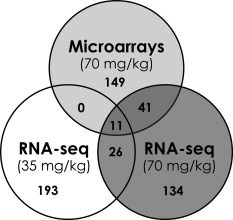
Venn diagram showing the number of genes in the hippocampus of BaP‐exposed Muta^™^Mouse that were differentially expressed (false‐discovery rate‐adjusted *P*‐values ≤ 0.05 and fold change ≥ ± 1.5 relative to control) using RNA‐seq and Agilent microarrays.

BaP did not affect gene expression at the lowest dose, but altered the expression of 230 and 212 genes (FDR *P* ≤ 0.05 and fold change ≥ ± 1.5) at the medium and high doses, respectively, 24 hr after the last treatment (Fig. 4 and Supporting Information Table 1). Planar cell polarity (PCP) was the top IPA pathway affected at the medium dose of BaP in the hippocampus. Six out of 63 genes that belong to this signaling pathway were downregulated and the pathway was predicted to be inhibited by the treatment (Z‐score of −2.4). “Neurological disease” was the top “diseases and disorders” category at the medium dose, with 26 genes associated with this category perturbed by BaP treatment. Transcript changes for the high dose of BaP were significantly enriched in genes under the control of estrogen receptor 1 (ESR1), the top upstream regulator in this dataset. The expression of 22 out of 30 ESR1‐regulated genes affected in this study was consistent with ESR1 activation (Z‐score of 3.5). In contrast, there was no single prominent upstream regulator identified in the genes that were altered at the medium dose. “Phospholipases” was the top IPA canonical pathway affected by the high BaP dose, reflecting the downregulation of endothelial lipase (*Lipg*) and upregulation of three phospholipases A2 (*Plag3*, *Plag4e*, and *Plag5*). Cancer was the most enriched category under “diseases and disorders” at the high dose, with a total of 179 molecules from that category perturbed.

#### Gene Expression Changes in the Hippocampus by DNA Microarrays

Microarray analysis revealed 259 differentially expressed transcripts (FDR *P* ≤ 0.05 and fold change ≥ ± 1.5) in the hippocampus following administration of the high dose of BaP relative to control mice (Supporting Information Table 2). Based on gene symbol, 52 of these genes were also identified as differentially expressed by RNA‐seq at the high dose (Supporting Information Table 3). As in the RNA‐seq IPA analysis, the results suggest that ESR1 was the top upstream regulator of differentially expressed genes. The top network for the shared gene set was “behavior, developmental disorder, and hereditary disorder”. The top affected IPA canonical pathway was “Axonal guidance signaling”, encompassing 12 genes including *Fzd4*, *Wnt3*, and *Wnt4*. These three genes are also part of the PCP pathway that was identified as perturbed in the RNA‐seq analysis of the medium and high doses.

#### Meta‐Analysis to Identify Diseases and Treatments with Similar Transcript Profiles in NextBio

**Figure 5:**
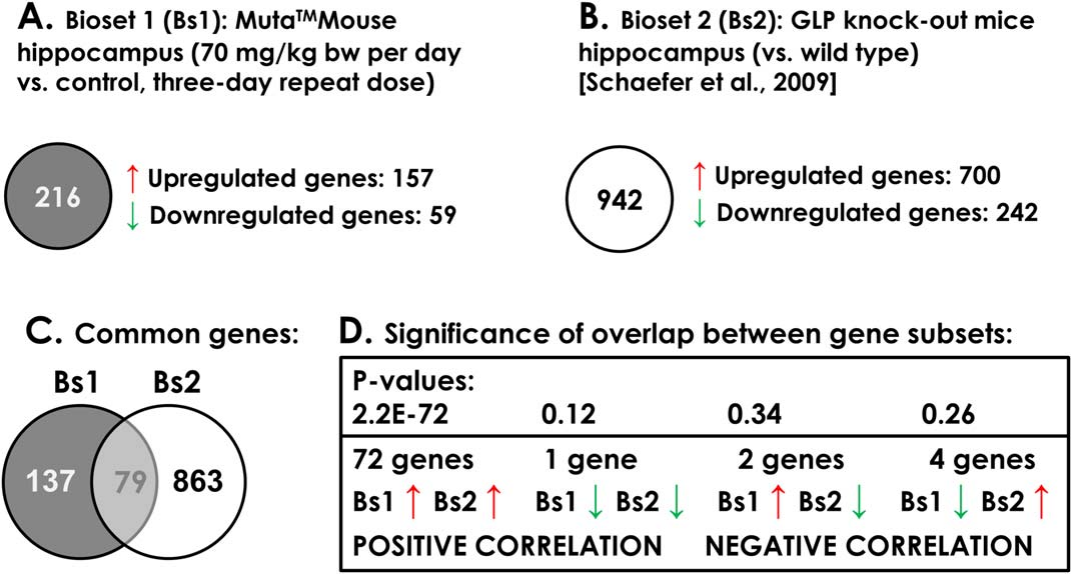
Identification of the most similar toxicogenomics study in NextBio. The gene expression profiles from the 70 mg/kg bw per day dose measured by RNA‐seq (bioset 1) compared with concurrent solvent controls was queried against the NextBio database. The analysis revealed that hippocampal gene expression from a GLP knock‐out study in mouse ([Schaefer et al., 2009], bioset 2) shared the greatest overlap with the BaP dataset. **A, B**: Characterization of each bioset. **C**: Number of genes that were differentially expressed in both data sets (false‐discovery rate‐adjusted *P*‐values ≤ 0.05 and fold change ≥ ± 1.5). **D**: Statistical significance of the overlap between the four gene subsets, determined by NextBio. We note that Next‐Bio and IPA count the number of differentially expressed genes differently; hence, the number of differentially expressed genes reported here is 216 vs. 212 in Fig. 4.

Query of the BaP‐exposed mouse hippocampal gene expression profiles (both RNA‐seq and microarray results) against the studies curated in NextBio revealed the most similar profiles were from the hippocampus of C57Bl/6 mice harboring postnatal, neuron‐specific ablation of Euchromatic Histone‐lysine N‐Methyltransferase 1 (also known as G9a Like Protein or GLP) compared to wildtype mice [Schaefer et al., 2009]. A comparison between profiles of mice with GLP ablation [Schaefer et al., 2009] and profiles from our study is shown in Figure 5. There were 79 genes in common between the two datasets (Fig. 5C; Supporting Information Table 4); of these, 72 were upregulated in both studies (Fig. 5D). The results are again consistent with activation of ESR1, which is supported by increased expression in both datasets in 15 of these 72 genes. In addition, the analysis revealed that BaP‐induced hippocampal gene expression shares much stronger similarity to compounds with “unclassified toxicity” (250 studies; the top similar category) than other toxicity types including “mutagens” (11 studies; the 19^th^ similar category). The “mutagens” category included three BaP studies in Muta^TM^Mouse lung and liver [Halappanavar et al., 2011], rat primary hepatocytes [Doktorova et al., 2013], and mouse hepatoma Hepa‐1c1c7 cells [Ovesen et al., 2014].

#### Validation of Gene Expression Changes by RT‐PCR

Twelve genes were selected for additional analysis by RT‐PCR. We purposefully selected genes that have previously been documented to be affected in the hippocampus following BaP exposure, albeit by different routes of exposure (gestational exposure [Brown et al., 2007] or intraperitoneal injection [Grova et al., 2007; Grova et al., 2008; Qiu et al., 2011; Cheng et al., 2013]). Most notably, the expression of NMDAR subunits including *Grin1*, *Grin2a*, and *Grin2b*, and the related genes *Grina* and *Grin3a* were examined. Finally, a selection of other genes identified in the RNA‐seq and microarray analysis were also included: *Cldn2* and *Cldn5*, ephrin type‐A receptor 7 (*Epha7*), hypoxia‐inducible factor 3A (*Hif3a*), 3‐hydroxy‐3‐methylglutaryl‐CoA synthase 2 (*Hmgcs2*), perilipin 4 (*Plin4*), semaphorin‐3B (*Sema3b*), and semaphorin‐5A (*Sema5a*). As Table 1 illustrates, no significant changes in the expression of any of these genes were observed using RT‐PCR for the low and medium doses. At the high dose, significant alterations occurred in the expression of six of the 12 genes (Table 1) in BaP‐exposed relative to control mice. The expression of nine of the 12 genes examined by RT‐PCR was consistent across at least two technologies (Table 1). In addition, the direction of change for affected genes was consistent, and the magnitude of change varied less than two‐fold, across all technologies.

**Table 1:** The Expression of Selected Genes Measured by RNA‐sequencing (RNA‐seq), Agilent Microarrays, and RT‐PCR, in the Hippocampus of Muta^™^M ouse 24 hr Following the Exposure to BaP for 3 Days

Accession number	Gene Symbol	Gene Name	RNA‐seq				RT‐PCR				**Microarrays**	
			35.0 mg/kg bw per day		70.0 mg/kg bw per day		35.0 mg/kg bw per day		70.0 mg/kg bw per day		70.0 mg/kg bw per day	
			Fold change	FDR p‐value	Fold change	FDR p‐value	Fold change	FDR p‐value	Fold change	FDR p‐value	Fold change	FDR p‐value
NM_008170	Grin2a	Glutamate receptor, ionotropic, NMDA2A (epsilon 1)	−1.60	0.329	1.22	0.763	1.22	0.609	**2.20**	**0.026**	**1.63**	**0.000**
NM_008169	Grin1	Glutamate receptor, ionotropic, NMDA1 (zeta 1)	−1.04	0.932	1.09	0.747	−1.00	0.766	1.52	0.111	1.33	0.000
NM_023168	Grina	Glutamate receptor, ionotropic, N‐methyl D‐aspartate‐associated protein 1 (glutamate binding)	1.07	0.860	1.03	0.916	1.13	0.621	**1.78**	**0.033**	**1.87**	**0.000**
NM_001033351	Grin3a	Glutamate receptor ionotropic, NMDA3A	−1.17	0.769	−1.46	0.128	1.03	0.748	1.15	0.358	−1.10	0.974
NM_013805	Cldn5	Claudin 5	**−2.15**	**0.000**	**−1.89**	**0.000**	−1.69	0.057	−1.49	0.118	−1.41	0.000
NM_016675	Cldn2	Claudin 2	2.21	0.210	**3.21**	**0.026**	2.49	0.103	1.86	0.163	1.30	0.000
NM_010141	Epha7	Eph receptor A7	1.31	0.432	**1.69**	**0.019**	1.20	0.836	**2.17**	**0.030**	**1.54**	**0.000**
NM_016868	Hif3a	Hypoxia inducible factor 3, alpha subunit	1.45	0.263	1.66	0.063	1.75	0.053	**2.96**	**0.004**	**1.63**	**0.000**
NM_008256	Hmgcs2	3‐hydroxy‐3‐methylglutaryl‐Coenzyme A synthase 2	−1.06	0.924	**−1.58**	**0.004**	−1.09	0.704	−1.00	0.925	**−1.61**	**0.000**
NM_020568	Plin4	Perilipin 4	**2.51**	**0.000**	**2.26**	**0.000**	1.40	0.139	**2.14**	**0.006**	**1.73**	**0.000**
NM_009153	Sema3b	Sema domain, immunoglobulin domain (Ig), short basic domain, secreted, (semaphorin) 3B	1.60	0.372	1.88	0.134	1.28	0.495	1.45	0.260	**1.57**	**0.000**
NM_009154	Sema5a	Sema domain, seven thrombospondin repeats (type 1 and type 1‐like), transmembrane domain (TM) and short cytoplasmic domain, (semaphorin) 5A	−1.23	0.416	**1.51**	**0.048**	1.02	0.847	**1.91**	**0.017**	**1.50**	**0.000**
NM_010368	Gusb	Glucuronidase, beta	1.05	0.929	1.05	0.894	1.00	1.000	1.00	1.000	1.07	0.872

Values that meet the ±1.5‐fold change and false‐discovery rate‐ (FDR) *P*‐value < 0.05 cut‐off are in **bold**.

Our RT‐PCR analysis did not examine changes in the expression of genes commonly associated with a DNA damage response. Therefore, another PCR array was used to measure the changes in the expression of genes that are directly or indirectly associated with BaP‐induced DNA damage and DNA damage response, such as the p53‐mediated DNA damage response gene cyclin‐dependent kinase inhibitor 1A (*Cdkn1a*), and other known BaP‐inducible genes such as NAD(P) quinone oxidoreducase 1 (*Nqo1*), cytochrome P450 1a1 (*Cyp1a1*), and cytochrome P450 1b1 (*Cyp1b1*). Previous work showed that hepatic expression of these genes was significantly altered by BaP exposure four and/or 24 hr following a three‐day repeat dose exposure to 5, 50, 150, or 300 mg/kg bw [Moffat et al., 2015] (see *Cyp1a1* summary in Supporting Information Fig. 2). Despite the fact that the aforementioned experiment covered the range of BaP doses used in this study (i.e., 5‐300 vs. 1‐70 mg/kg bw per day), our RT‐PCR array analysis did not reveal significant changes in any of these genes in the hippocampus of BaP‐treated mice relative to controls (Supporting Information Table 5). However, we note that some genes involved in xenobiotic metabolism of BaP that were not on our PCR arrays were identified in the BMD analysis described below.

#### Benchmark Dose (BMD) Analysis of Transcriptional Changes

The genomics data generated here were used for dose‐response modeling and BMD determination, as was conducted for other tissues from BaP‐exposed animals examined previously [Moffat et al., 2015]. To this end, the dose‐response data for all 387 mouse IPA pathways were modelled using BMDExpress. First, we identified 11 IPA pathways containing *Grin1*, *Grin2a*, *Grin2b, Grin2c, Grin2d, Grina*, and *Grin3a* (Supporting Information Table 6), and investigated median BMD values for these pathways. We also identified the ten “most sensitive” pathways (i.e., those with the lowest median BMDLs) (Supporting Information Table 7). This analysis identified that three out of the 11 IPA pathways that include the Grin proteins (Amyotrophic Lateral Sclerosis Signaling, Synaptic Long Term Potentiation, and Huntington's Disease Signaling) were also among the ten most sensitive pathways (Supporting Information Table 7). The most sensitive pathways were “Synaptic Long Term Potentiation” and “G‐Protein Coupled Receptor Signaling”, both yielding median BMDL values of 5.2 mg/kg bw per day. Other IPA pathways with low BMDLs included “Xenobiotic Metabolism Signaling” and “Nrf2‐mediated Oxidative Stress Response” (Supporting Information Table 7). These two pathways include several genes involved in BaP metabolism. For example, the expression of epoxide hydrolase 1 (*Ephx1*) and aldehyde oxidase 1 (*Aox1*) was increased by 1.7‐ and 1.6‐fold, respectively, at the high dose, and glutathione *S*‐transferase mu 2 (*Gstm2*) was increased by 2.4‐fold at the medium dose. These two IPA pathways were also affected in the lungs and livers of BaP‐treated mice, and were considered as a part of a toxicogenomics‐based carcinogenic MOA developed for BaP [Moffat et al., 2015]. Consistent with the lack of BaP effect on DNA damage‐inducible genes, the IPA pathways that were used in deriving toxicogenomic PODs for mouse liver, lung, and forestomach (so selected because of their relationship to the key event “DNA adducts and DNA damage” in the carcinogenic MOA for BaP developed previously [Moffat et al., 2015]), were either unaffected (Supporting Information Fig. 3) or contained only 2 or 3 affected genes per pathway (data not shown).

## DISCUSSION

Measurement of genome‐wide transcriptomic responses to BaP permits an unbiased evaluation of potential MOAs and modulating factors involved in its neurotoxicity in adult rodents. Our study confirms expression changes in NMDARs and suggests that ESR1 activation occurs following BaP exposure, which may be involved in the learning and memory processes that are regulated by GLP in the brain. Our analyses do not support extensive DNA damage responses or alterations in cell cycle (typical transcriptional consequences of genotoxicity) in the hippocampal tissue.

Prior to whole‐genome profiling we measured BaP‐DNA adduct formation in order to gain insight into the distribution of BaP and/or its metabolites in the brains of treated animals. Cerebellum was used as a surrogate region for adduct analyses due to the prohibitively small size of the murine hippocampus. Metabolised BaP did reach the brain, as indicated by the presence of dG‐*N*2‐BPDE adducts measured by ^32^P‐post‐labeling (Fig. 2). This result is consistent with a previous report of increased levels of BaP‐DNA adducts in rat cortex following one‐, two‐, or three‐month exposures to BaP by intraperitoneal injection [Nie et al., 2013]. However, the magnitude of the cerebellar DNA damage observed in this study was 2‐3 times lower than that previously observed in Muta^TM^Mouse lung and liver (Fig. 3) [Halappanavar et al., 2011]. Closer inspection of the interpolated values for the BaP‐DNA adducts data from previous studies shows that there were 116.5 ± 72.8 adducts per 10^8^ nucleotides in the liver 24 h after the last BaP exposure at the interpolated dose of 70 mg/kg bw per day, which overlaps with the cerebellum adduct level of 64.2 ± 17.6 adducts per 10^8^ nucleotides at this dose from this study (Fig. 3). Therefore, these differences in BaP‐DNA adduct levels between the cerebellum, liver and lung may be considered relatively small.

Our data suggest that the transcriptional DNA damage response is not as prevalent in the brain as in other tissues. For example, IPA pathways relating to DNA damage response and cell cycle alterations used in assessing the carcinogenic MOA of BaP [Moffat et al., 2015] were only marginally affected in the hippocampus. Overall, the DNA damage response commonly associated with BaP does not appear to be a significant driver of BaP‐induced neurotoxicity based on our data. Thus, our study does not support that BaP‐induced genotoxicity leading to neuronal cell death is the most plausible MOA (reviewed in [Chepelev et al., 2015]). Nevertheless, additional studies are required to fully support this conclusion, such as transcriptional profiling of the hippocampus of wild type and DNA repair‐deficient mice following BaP treatment.

We set out to evaluate the evidence to support the hypothesis that transcriptional perturbations of NMDARs may be associated with impaired learning and memory (MOA reviewed in [Chepelev et al., 2015]). Briefly, permeability to calcium confers NMDARs the ability to participate in increasing synaptic signalling (i.e., long‐term potentiation), which is the current model for learning and memory formation in the hippocampus. Transgenic rodent models with hippocampus‐specific alterations of NMDR expression confirm the importance of this receptor in learning and memory. For example, knock‐out of NR1 expression in the murine CA1 hippocampal region compromises their performance in the Morris water maze test [Shimizu et al., 2000], while forebrain‐specific overexpression of NR2B in aged mice enhances their performance in five different memory tests compared to age‐matched wild type controls [Cao et al., 2007]. Thus, NMDARs are ideal hypothetical candidates for involvement in the neurotoxicity of BaP.

Given the established importance of NMDARs in learning and memory, and previous publications suggesting that NDMARs may be perturbed following BaP exposure [Brown et al., 2007; Grova et al., 2007; Grova et al., 2008; Qiu et al., 2011; Cheng et al., 2013], we specifically investigated the response of NMDAR transcripts in order to explore their potential role in the neurotoxic effects of BaP. There have been five studies on the effects of BaP on NMDAR in the hippocampus of adult mouse or rat hippocampus, each of them reporting perturbed expression of at least one of the three subunits *Grin1*, *Grin2a*, or *Grin2b* (Table 2). However, the published results are inconsistent. Our results demonstrate measurable increases in *Grin2a* by both RT‐PCR and DNA microarrays, in addition to increased expression of *Grina*, another NMDAR (Table 1). The direction of the noted changes of *Grin2a* in the hippocampus of mice and rats in response to BaP differs between studies (Table 2) and the differences could be sex‐specific (i.e., three studies support increases in adult males, but the single study on BALB/c adult female mice shows decreased expression). Alternatively, the noted difference could arise due to different timing between the last treatment and the necropsy. Nonetheless, regardless of the direction of change, all five published studies show altered expression of at least one of the NMDAR genes in rodent hippocampus (Table 2). It is possible that altered expression of *Grin1*, *Grin2a* and/or *Grin2b* may be sufficient to change the molecular composition of NMDARs, thereby interfering with the normal function of this type of glutamate receptor. Therefore, our data confirm previous reports demonstrating altered expression of NMDARs following BaP exposure, supporting the potential involvement of NMDARs in mediating BaP neurotoxicity.

**Table 2:** The Effect of BaP on Gene Expression of NMDAR Subunits NR1 (Grin1) and NR2 (Grin2) in the Mouse and Rat Hippocampus

Animals	Exposure and duration	NR1	NR2A	NR2B	References
Long Evans rat ♀, ♂	Gestational exposure (oral gavage of mothers) at gestational days 14‐17; measured at postnatal days 2, 5, 10, 15, 20	N. A.	↓	↓	[Brown et al., 2007]
BALB/c mouse ♀, adult	Intraperitoneal injection for 10 days	↑	N. A.	N. A.	[Grova et al., 2007]
BALB/c mouse ♀, adult	Intraperitoneal injection for 10 days	↑	↓ (2 high doses)	↓	[Grova et al., 2008]
Sprague‐Dawley rat ♂, adult	Intraperitoneal injection for 98 days	N. A.	↑	N. A.	[Qiu et al., 2011]
Sprague‐Dawley rat ♂, adult	Intraperitoneal injection for 45 days		↑		[Cheng et al., 2013]
Muta^TM^Mouse ♂, adult	Oral gavage for 3 days	N. C.	↑	N. C.	[This study]

N. A., not available (not measured); N. C., no change.

It has been proposed that *Grin2a* is controlled by AHR since *Grin2a* levels decline following AHR silencing, and increase with AHR stimulation by 2,3,7,8‐Tetrachlorodibenzodioxin (TCDD) in rat cortical neurons [Lin et al., 2009]. Therefore, the *Grin2a* upregulation observed in our study may be due to AHR activation by BaP. However, other classical targets of AHR‐mediated transcriptional changes, including *Cyp1a1*, *Cyp1b1*, and *Nqo1*, were unaffected in the hippocampus following BaP exposure as measured by RNA‐seq, microarrays, and RT‐PCR. If both *Cyp1a1* and *Grin2a* are indeed transcriptional targets of AHR‐mediated BaP‐induced changes, the lack of an effect on *Cyp1a1* expression in the hippocampus 24 hr after the last exposure is similar to hepatic, but not pulmonary effects observed in Muta^TM^Mouse 24 hr after a 3‐day repeat oral gavage to similar BaP doses (50 and 150 mg/kg bw per day, Supporting Information Fig. 2) [Moffat et al., 2015]. It is unlikely that the lack of a BaP effect on the expression of *Cyp1a1* 24 hr after the last exposure in the hippocampus and liver is due to declining levels of BaP and its metabolites, since intraperitoneal injection of Wistar rats with radio‐labeled BaP showed a peak of radioactivity in the brain, lung, and kidney 23 hr after administration [Das et al., 1985]. Therefore, additional work is needed to determine whether *Grin2a* and other NMDAR genes are under the transcriptional control of AHR, for example, by exposing AHR knock‐out animals to BaP and conducting genomics and neurobehavioral experiments.

The presence of BaP metabolites in the brain was clearly indicated by the dose‐related increases in the frequency of BaP‐DNA adducts. Mono‐hydroxylated metabolites of BaP share strong structural similarity to the classical estrogen receptor ligand 17‐beta‐estradiol (E2). The mono‐hydroxylated BaP metabolites 3‐OH‐BaP and 9‐OH‐BaP, but not the parent compound, competitively bind to recombinant human ER alpha and ER beta receptors *in vitro* to strongly induce a beta‐galactosidase reporter gene in human MCF‐7 cells [Fertuck et al., 2001]. Although we have no evidence to support the presence of hydroxylated BaP metabolites in the brain, the presence and dose‐dependent increase of 3‐OH‐BaP in whole rat brain following single administration of 5, 50, or 100 mg BaP/kg bw by intragastrical administration has been reported 12 hr after administration [Liu et al., 2015]. Interestingly, that work revealed about 20, 55, and 75‐fold excess of 3‐OH‐BaP over (+)‐anti‐BPDE at 5, 50, and 100 mg/kg doses; the concentration of both metabolites was 456.2 ± 56.4 and 24.3 ± 1.3 at the 5‐mg/kg dose, respectively [Liu et al., 2015]. These results suggest the presence of mono‐hydroxylated BaP metabolites in the animals examined in our study and the reported estrogenic properties of these metabolites are consistent with robust activation of ESR1 predicted by our transcriptomic data. Indeed, ESR1 was identified as the top upstream regulator (in an activation state) in both the microarray and RNA‐seq analysis. Furthermore, of the 72 genes that are shared between this and the most similar study identified using NextBio, 15 are regulated by ESR1, again suggesting activation. These results, as well as the existence of established AHR‐ESR cross‐talk [Kummer et al., 2008], suggest the involvement of these receptors in mediating BaP neurotoxicity and merits further investigation.

According to an emerging concept, exposure to genotoxic compounds may cause both carcinogenicity and neurotoxicity, depending upon the presence or absence of active cell division [Kisby et al., 2011]. A previous study [Hakura et al., 1998] used the Muta^TM^Mouse model to support this concept. Five days of exposure to 125 mg BaP/kg bw per day led to high *lacZ* mutant frequencies in tissues prone to BaP‐induced carcinogenicity (e.g., forestomach, liver, lung) on the 14^th^ day after the last BaP treatment. However, increased mutant frequencies were not observed in the brain, prompting the authors to suggest the crucial role of rapid cell division in BaP carcinogenicity. In the case of MAM, there is a disruption of common signaling pathways in the colon and the brain (i.e., the TGF‐beta, Wnt, and MAPK pathways), leading to either carcinogenicity or neurotoxicity, respectively [Kisby et al., 2011]. Whether these pathways are also shared between the tissues exhibiting BaP‐mediated carcinogenicity and neurotoxicity is unknown. A comparison of 24 hr post‐exposure liver transcriptomic profiles in response to 300 mg BaP/kg bw per day for three days [Moffat et al., 2015] and the 24 hr post‐exposure hippocampal gene expression in response to 70 mg BaP/kg bw per day for three days (i.e., this study), indicates very little effect (Fischer's exact *P*‐values of 1.0 for liver and 0.08 for hippocampus) on the Wnt/catenin signaling pathway in these tissues; however, it is worth noting that there were three (*Ccnd1*, *Cdh1*, and *Wnt5b*) and four (*Fzd4*, *Sfrp5*, *Wnt7a*, and *Wnt7b*) genes affected from this pathway in liver and hippocampus, respectively. Toxicogenomic studies provide “snapshot” representations of dynamic gene expression networks; thus, despite the lack of support for the aforementioned hypothesis [Kisby et al., 201] in our study, the effects of BaP on common signaling pathways that would lead to either neurotoxicity or cancer do merit further investigation.

A meta‐analysis of publicly available data revealed that the gene expression pattern that is most similar to the one we describe herein was found in the hippocampus of GLP knock‐out mice compared to wild type [Schaefer et al., 2009]. Again, genes shared between the GLP knock‐out study and this study are indicative of ESR1 activation. GLP is a histone‐lysine N‐methyltransferase; neuron‐specific, postnatal knock‐out of GLP in mice leads to transcriptional de‐repression of many non‐neuronal and neuron progenitor genes in adult neurons, as well as complex behavioral abnormalities [Schaefer et al., 2009]. For example, in an elevated plus maze test that assesses awareness of potential danger or anxiety by measuring the degree to which the rodent avoids the open arm of the maze, GLP knock‐out mice showed an increased number of entries into open arms, spent more time in open arms, and had lower total arm entries [Schaefer et al., 2009]. Very similar behavioral outcomes on the elevated plus maze test were observed in adult mice treated with BaP [Bouayed et al., 2012], suggesting that animals with both genotypes had reduced awareness of potential danger. Due to the different tests used in the two studies, further direct comparison of the array of possible neurotoxic effects is impossible. However, many of the aforementioned studies of BaP neurotoxicity in adult rats and mice (e.g., [Grova et al., 2007; Cheng et al., 2013; Qiu et al., 2011]) characterized the neurotoxic effects of BaP administration as defects in learning, as measured by deficits in spatial learning in the Morris water maze test, which is comparable to impaired ability of the GLD knock‐out mice to remember negative experiences in the fear conditioning paradigm [Schaefer et al., 2009]. Due to the similarity of GLP knock‐out‐mediated neurotoxicity and BaP‐induced alterations in neurobehavioral task performance related to learning and memory, as well as similar effects on ESR1‐mediated gene expression between the studies, the involvement of ESR1 in BaP‐induced neurotoxicity also warrants further investigation.

BMD analysis revealed that three out of the 11 IPA pathways in which Grin proteins are involved (Amyotrophic Lateral Sclerosis Signaling, Synaptic Long Term Potentiation, and Huntington's Disease Signaling) were also among the 10 “most sensitive pathways” (i.e., those with the lowest median BMDL values). The Synaptic Long Term Potentiation pathway had the lowest median BMDL (5.2 mg/kg bw per day) and is biologically relevant based on the proposed role of NMADRs in the neurotoxic MOA of BaP. Thus, we propose that this is the most relevant pathway on which to derive a toxicogenomics point of departure (POD) estimation for adult neurotoxicity following BaP exposure. This POD is in consistent with PODs of 1.0, 3.7, and 7.4 mg/kg bw per day that we determined using liver, lung, and forestomach toxicogenomics profiles, respectively, for cancer risk assessment [Moffat et al., 2015]. Therefore, the toxicogenomics PODs for adult mouse neurotoxicity and carcinogenicity are very similar. It remains to be determined whether toxicogenomics PODs would be significantly lower should exposures be conducted during critical developmental stages.

In conclusion, our study provides experimental data that support an NMDAR‐mediated MOA for BaP‐induced hippocampal responses and concomitant neurotoxicity. We identified a number of genes regulated by ESR1 that are potentially important in mediating the neurotoxicity of BaP and other PAHs other PAHs, as well as several neurotoxic compounds and conditions leading to neurotoxic outcomes (e.g., GLP knock‐out in the mouse discussed above). Although DNA adduct levels could not be measured in the hippocampus of the animals examined, our hippocampal gene expression analysis, which employed three different technologies, provides little support for significant DNA damage in this tissue. However, these results should be interpreted with caution and we emphasize that further studies are needed to conclusively determine whether genotoxicity plays a role in the altered learning and behavior observed following rodent BaP exposures. The efficiency of DNA repair in the human brain is age‐dependent, such that age‐dependent increases in the extent of oxidative DNA damage appears to reduce the expression of certain genes involved in learning, memory, and neuronal survival [Lu et al., 2004]. Therefore, it is conceivable that the timing of BaP exposure is a crucial factor that determines whether BaP induces neurotoxic effects, and moreover, the MOA by which the effects are manifested (i.e., genotoxic vs. NMDAR‐dependent). Transcriptomics studies similar to this one in DNA repair‐deficient mice, in younger exposed animals, and in animals examined at various times post‐exposure would provide additional evidence to assess the extent to which BaP genotoxicity plays a role in its neurotoxicity in developing and adult rodents. In addition, the extent to which DNA damage‐induced cytotoxicity during critical periods of brain development (i.e., during periods of extensive cellular proliferation) contributes to neurotoxicity should be studied in detail.

## AUTHOR CONTRIBUTIONS

CLY, PAW, and NLC conceived of the idea for the work and designed the experiments. WJB aided in experimental design and interpretation of results. ASL conducted the animal work. DHP and VMA conducted the experiments to determine BaP adducts, and analyzed and interpreted these data. NLC and RG performed the global transcriptional analysis. RG, AW, and BK analyzed the data. NLC drafted the manuscript and all authors approved the final manuscript.

## Supporting information

Supporting Information

Supporting Information

Supporting Information

Supporting Information

## Footnotes

1 CAS no. 50‐32‐8, now also known as benzo[*pqr*]tetraphene.
