# Supplementary figures and images for: Transcriptional profiling of the mouse hippocampus supports an NMDAR‐mediated neurotoxic mode of action for benzo[a]pyrene
Source: Environ Mol Mutagen. 2016 May 19;57(5):350–63. doi: 10.1002/em.22020 (PMC4915531; doi:10.1002/em.22020)

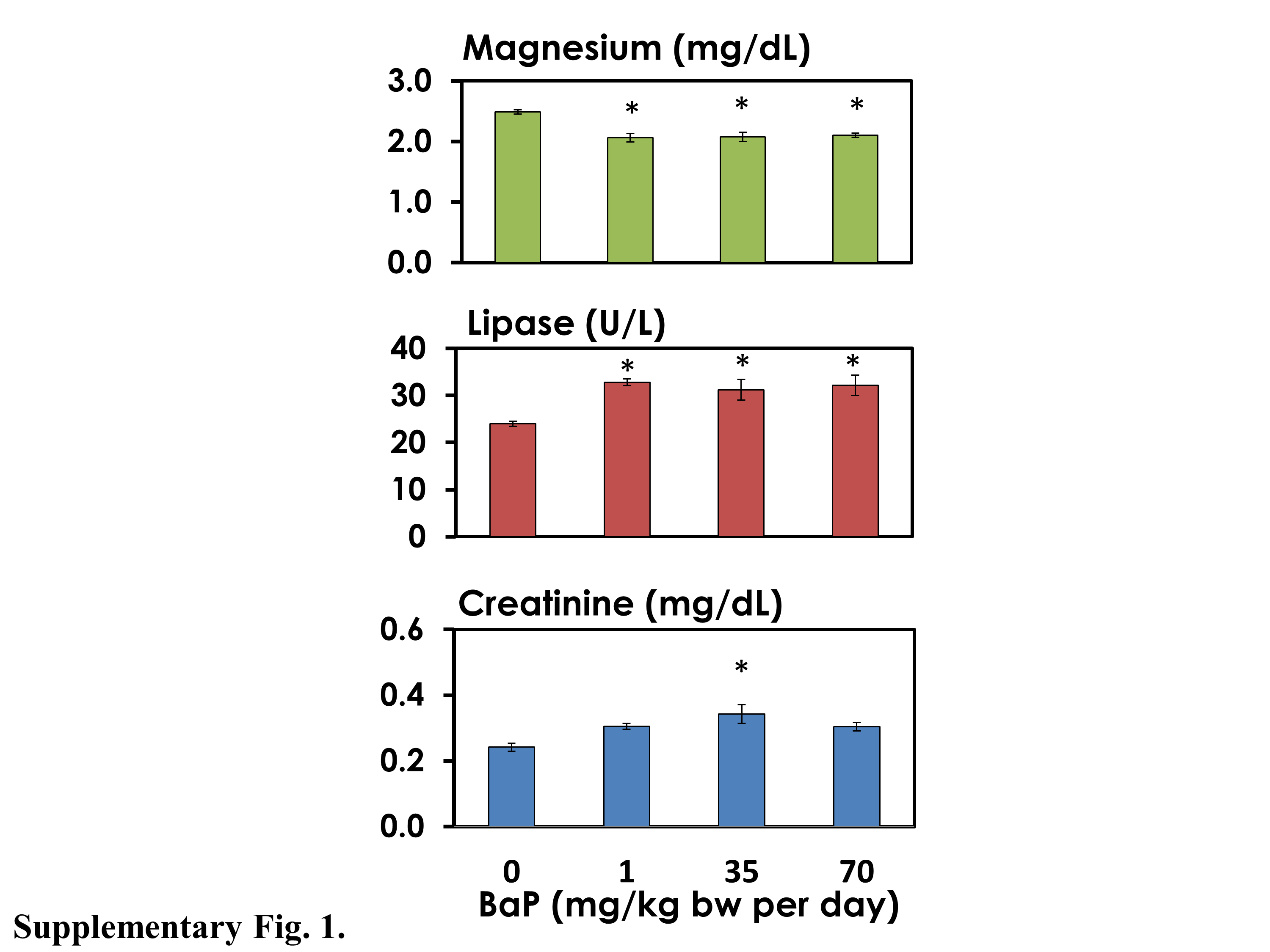

Supplement: Supplementary file 1 — Supporting Information [file EM-57-350-s001.tif]

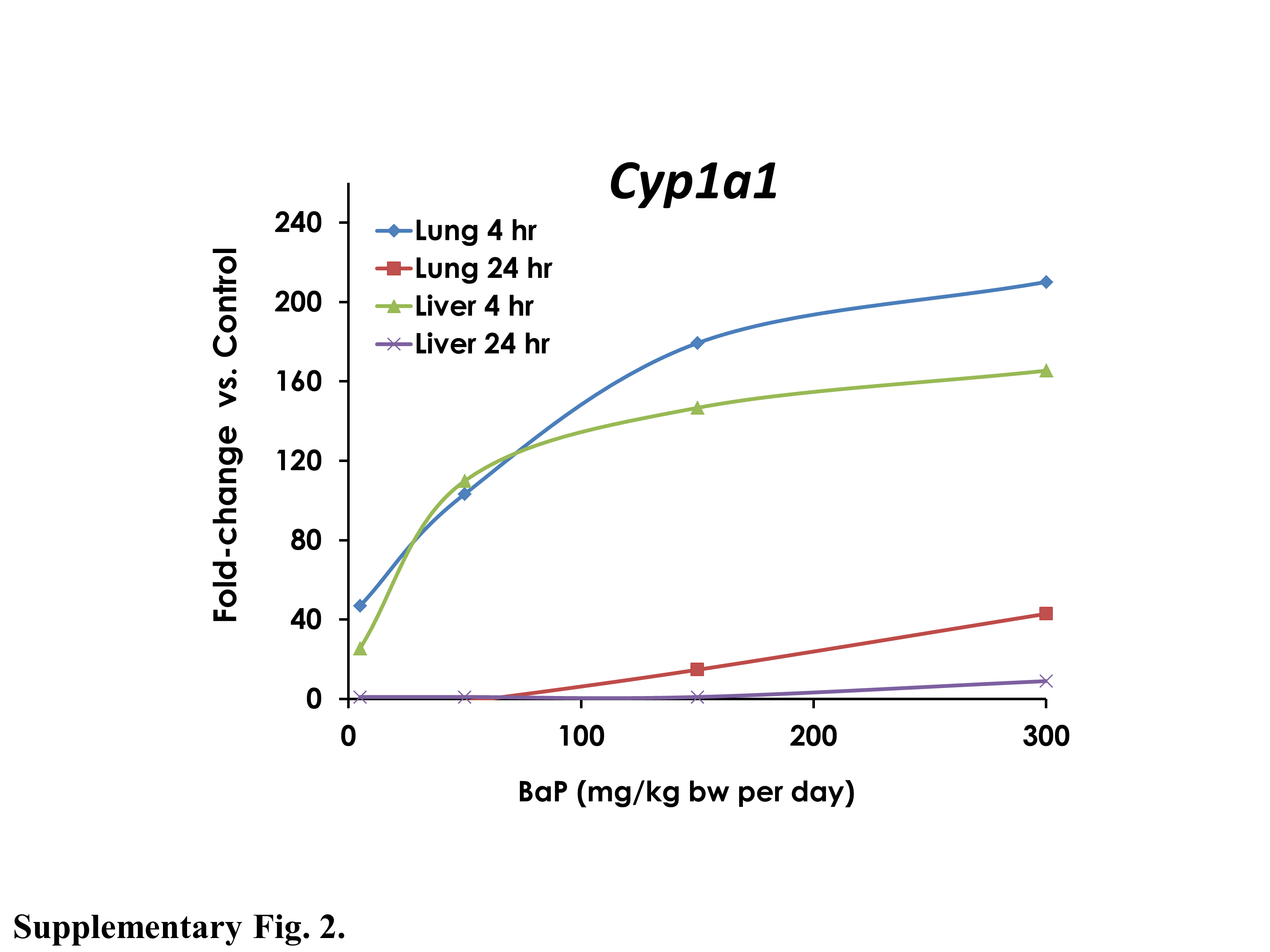

Supplement: Supplementary file 2 — Supporting Information [file EM-57-350-s002.tif]

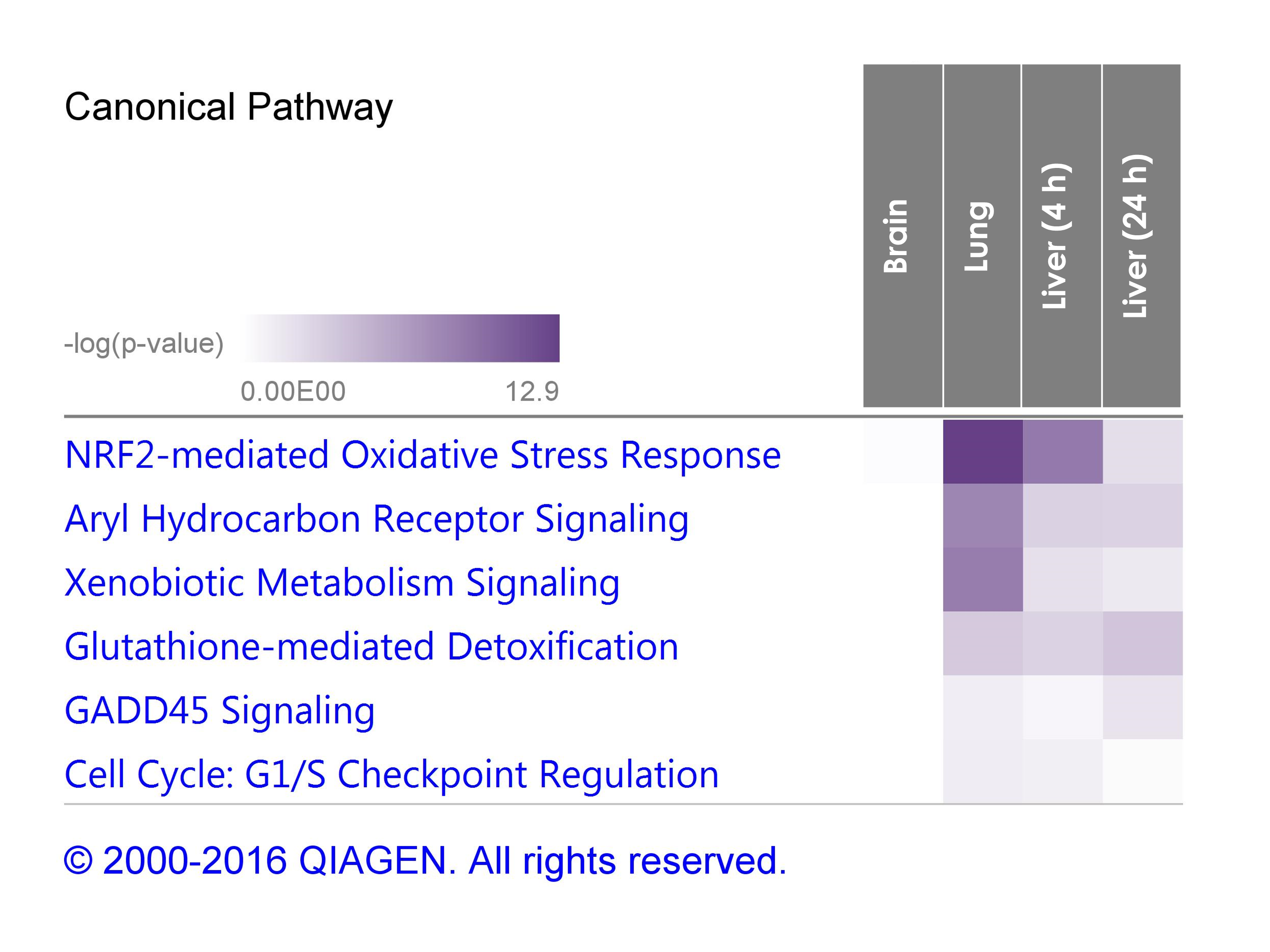

Supplement: Supplementary file 3 — Supporting Information [file EM-57-350-s003.tif]
